# Revisiting the Metallo-*β*-Lactamase–Mediated Antibiotic Resistance: Exploring Novel Mechanisms and Therapeutic Strategies

**DOI:** 10.1155/ijm/1574819

**Published:** 2025-10-28

**Authors:** Jahanvi Saini, Silvi Gautam, Divakar Sharma, Ankit Khanduri, Divya Venugopal

**Affiliations:** ^1^Department of Microbiology, Graphic Era (Deemed to Be) University, Dehradun, India; ^2^Department of Biotechnology, Graphic Era (Deemed to Be) University, Dehradun, India; ^3^Department of Microbiology, Graphic Era Institute of Medical Sciences, Dehradun, Uttarakhand, India

**Keywords:** *β*-lactamase, *β*-lactams, antimicrobial resistance (AMR), Enterobacteriaceae, metallo-*β*-lactamase

## Abstract

*β*-Lactam resistance is one of the major health concerns today, primarily due to enzymes called *β*-lactamases. Metallo-*β*-lactamases (M*β*Ls) can cleave a wide range of *β*-lactam antibiotics, including carbapenems. These enzymes require zinc (Zn) ions to function and can be inhibited by conventional *β*-lactamase inhibitors. According to their structural and functional characteristics, M*β*Ls are categorized into three classes known as B1, B2, and B3, each with distinct substrate preferences and resistance mechanisms. The prevalence of acquired M*β*Ls, such as IMP, VIM, and NDM, demonstrates the necessity for the development of effective treatments. Novel therapeutic approaches have emerged as potential treatment options. However, antibiotic toxicity, resistance development, and coexisting resistance mechanisms, such as efflux pumps and porin modifications, complicate treatment strategies. Recent advances in diagnostics have significantly improved the rapid identification of M*β*L-producing bacteria, suggesting the selection of treatment and antimicrobial stewardship. This review highlights the urgent need for global efforts to combat M*β*L-mediated resistance through surveillance, advanced diagnostics, and innovative therapies, emphasizing the role of advanced and innovative theragnostic approaches in managing M*β*L infections.

## 1. Introduction

Antibiotics are regarded as a breakthrough of the 20th century; however, the emergence of antimicrobial resistance (AMR) signals a troubling phase for the antibiotic era [1]. Many Gram-negative bacteria produce various complex *β*-lactamases, often encoded by plasmids, which are the main drivers of antibiotic resistance [2]. The presence of these enzymes poses a significant public health risk and complicates treatment, whether acquired in the community or healthcare settings. Horizontal gene transfer (HGT) between species leads to the spread of drug-resistant infections [3]. The sudden growth of antibiotic resistance observed in pathogens such as *Acinetobacter baumannii*, *Klebsiella pneumoniae*, *Mycobacterium tuberculosis*, and *Escherichia coli* has shown resistance to new generations of therapeutics [4, 5]. Almost 2800 different *β*-lactamase enzymes have been well studied and emerged from the environment, defending microorganisms against naturally occurring antibiotics [6]. These enzymes seem to have originated from penicillin-binding proteins (PBPs) due to their shared sequence similarities with *β*-lactamases, including a catalytic serine residue in the active site. The first *β*-lactamase was recognized in *E. coli* before the commercial introduction of penicillin [7]. As pharmaceutical industries produced modified *β*-lactam drugs, however, resistance developed against almost every drug, which is due to the high mutation rate and increasing efficiency in making antibiotics ineffective [8].

*β*-Lactamases share common resistant mechanisms, that is, chromosomally mediated and plasmid-mediated mechanisms, which break four-membered rings of *β*-lactam antibiotics [9]. The enzymes are categorized according to their mechanisms, either by forming an acyl-enzyme complex with a serine residue at the active site or by promoting a hydrolytic reaction that involves one or two active Zn ions [7]. These enzymes inactivate antibiotics through hydrolysis of their *β*-lactam ring, a process facilitated by a zinc ion, which mediates antibiotic resistance. They escape the inhibition by traditional *β*-lactamase inhibitors, which makes treatment for infections more difficult. Since M*β*L genes are frequently carried on plasmids, bacterial populations can spread rapidly. Due to the ineffective treatments, M*β*L-producing bacteria pose a serious risk in healthcare environments [10].

According to the biochemical characteristics, *β*-lactamases have been classified as enzymes. Bush et al. suggested a classification scheme based on functional properties. This classification comprised three main subgroups based on their molecular weights, isoelectric points, and substrate and inhibitor profiles [11]. Based on the primary structure, an alternative classification was introduced in 1980. At that time, peptide sequencing was used in some laboratories for identification [12]. There are four classes of *β*-lactamases: A, B, C, and D. This classification system remains relevant even after three decades. Serine *β*-lactamases (S*β*Ls) belong to Classes A, C, and D, each exhibiting unique features, while metalloenzymes are Class B, which depend on Zn-metal ions. Class B is further subcategorized into three subclasses: B1, B2, and B3 [11].

## 2. Mechanism of *β*-Lactam Antibiotic Resistance

Most *β*-lactams, such as penicillin, are designed to block enzymes called PBPs, which help in building strong cell walls. The cell wall is an outer shield, giving bacteria their shape and protecting them in harsh situations, like in the bloodstream, lungs, or urine [4]. The wall is built from repeating units of two sugars, N-acetylglucosamine (NAG) and N-acetyl muramic acid (NAM). Another class of enzymes links these sugars and blocks together, but PBPs glue two NAM chains using a D-alanine–D-alanine bond, helping the wall to toughen [13]. Strominger discovered that the *β*-lactam ring in these antibiotics showed similarities to the D-alanine–D-alanine part of the cell wall. Due to this, the PBPs get tricked into treating the drug as if it were the building blocks they commonly handle, leading to a halt in cell wall construction and ultimately the death of the bacteria. Bacteria developed resistance; they found ways to stop the antibiotic from binding to their PBPs or break down the drug [13, 14].

**Figure 1:**
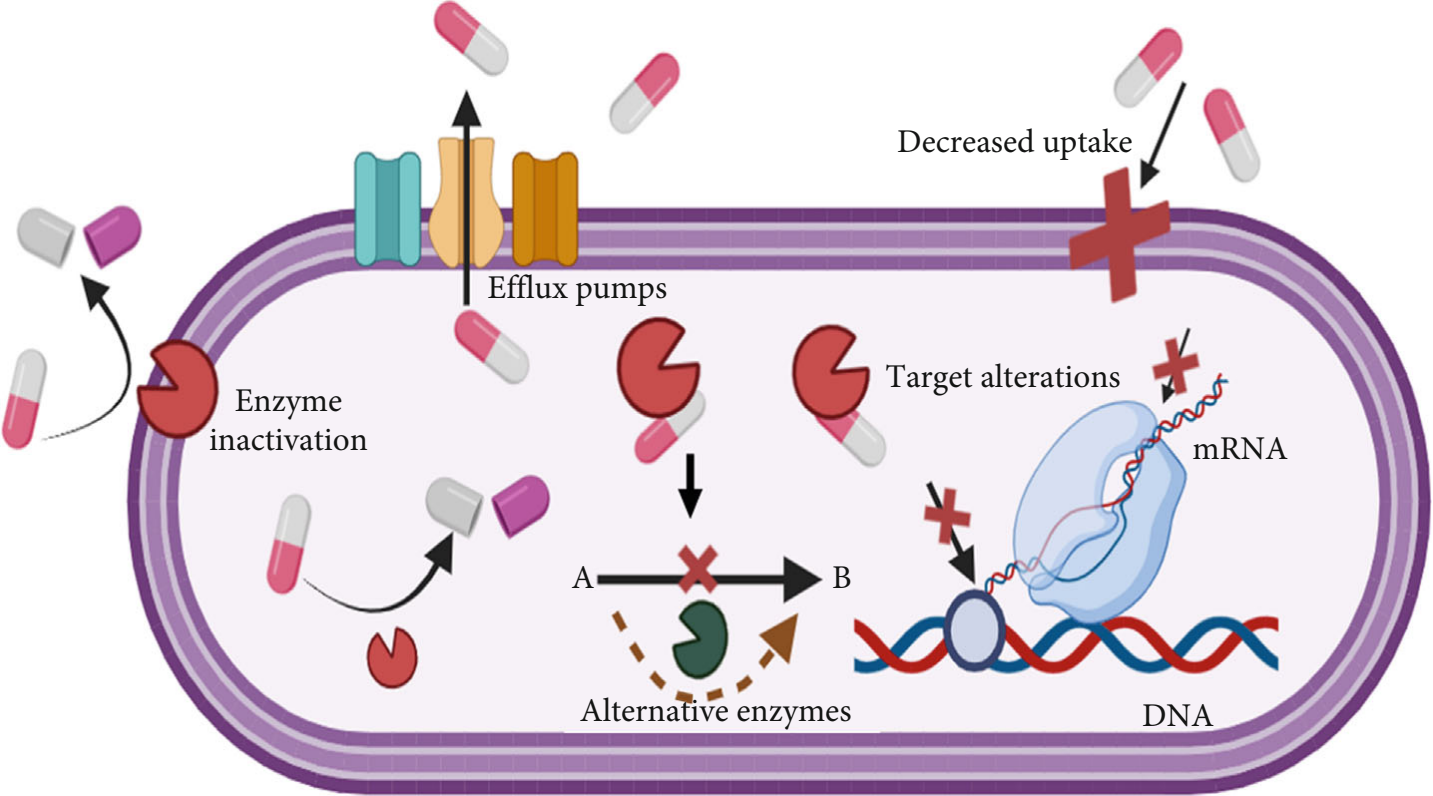
Diagrammatic presentation of antibiotic resistance mechanisms in bacteria: enzymatic inactivation via *β*-lactamases, overexpression of efflux pumps, reduced uptake of drugs by underexpressed porins, and target site modification.

Bacteria become resistant to *β*-lactam antibiotics through four main pathways (Figure 1):

o. Inactivation by enzymes: Bacterial enzymes interact with antibiotics and alter and render them ineffective against the bacteria. This modification happens through the transfer of genes responsible for antibiotic resistance via horizontal transfer of plasmids [15]. A well-known example is *β*-lactamases, which break down *β*-lactam antibiotics like penicillin and cephalosporin [6].
o. Overexpression of efflux pumps: These proteins can export a broad range of substances, such as antibiotics, that are released from the cell, and the bacteria overexpress these efflux pumps to push the antibiotics out of the cell [16]. This resistance mechanism has a vital role in *Acinetobacter* spp. and *Pseudomonas* spp. [16, 17].
o. Decreased expression of porins: The absorption is reduced because of the changes in porin expression or modifications in the outer membrane permeability. The changes decrease or block the entry of antibiotics [18].
o. Drug target modifications: Structural changes in the drug-specific ligands prevent the antibiotics from binding properly, thereby decreasing their effectiveness [4].

*β*-Lactamase-producing pathogens exhibit multilayered resistance mechanisms that work synergistically to confer high levels of antibiotic resistance. Efflux pumps (e.g., MexAB-OprM and AcrAB-TolC) actively expel *β*-lactams, reducing intracellular drug concentration, while mutations in porin limit antibiotic entry, further decreasing susceptibility [19]. Additionally, coproduction of S*β*Ls and M*β*ls (e.g., KPC and OXA-48) hydrolyzes *β*-lactams, leading to near-complete resistance against carbapenems and cephalosporins [20]. Target modifications, such as 16S rRNA methylation, reduce aminoglycoside binding, making the therapy ineffective [21].

## 3. Overview of *β*-Lactamases

**Figure 2:**
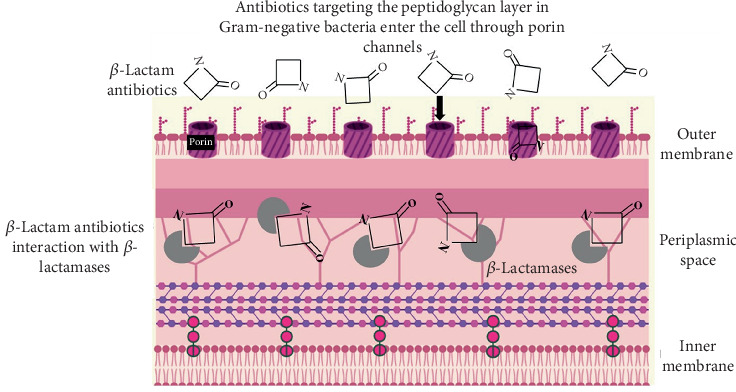
Illustration of the enzymatic hydrolysis of the *β*-lactam ring by *β*-lactamases, rendering the antibiotic ineffective.

*β*-Lactamases are the enzymes produced by bacteria and have the capacity to cleave chemical compounds that contain a *β*-lactam ring (Figure 2). The initial methods for classifying these enzymes were based on the quick hydrolysis of penicillin and early cephalosporins and reacted to agents that modify proteins [22, 23]. The introduction of new substrates and inhibitors in clinical settings, together with decreasing costs and increased standardization of gene sequencing, has led to an expanded classification system that combines both molecular and functional characteristics [11, 24].

Researchers have identified several functional groups, which are linked to the four molecular classes, as presented in Table 1 [24]. Enzymes were grouped according to their capacity to hydrolyze various *β*-lactam substrates, including monobactams, carbapenems, cephalosporins, and penicillin. They are characterized through their reactions to the broad-spectrum S*β*L inhibitor (avibactam), the class-A *β*-lactamase inhibitor (clavulanic acid), and the metal-binding agent EDTA, which is useful for identifying M*β*Ls [25, 26].

### 3.1. Classification of *β*-Lactamases

Classification systems available for categorizing *β*-lactamase enzymes are based on two principles, namely, the Ambler and Bush–Jacoby systems. The Ambler classification system groups *β*-lactamases into Classes A through D based on similarities in their amino acid sequences [27]. Conversely, the Bush–Jacoby system classifies these enzymes according to their ability to break down various substrates and their response to inhibitors [24, 28].

The arranged profiles emphasize structural resemblances among the S*β*Ls in Classes A, C, and D (Table 1). Additionally, Class B enzymes are different from others and are referred to as M*β*Ls. These enzymes typically contain one or two Zn^2+^ ions at their active sites. Table 1 presents a revised edition of the functional classification system initially proposed by Bush in 1989 and subsequently updated in 1995 [24, 28]. It integrates structural and functional classification as closely as feasible, using publicly available information. Significant expansion in *β*-lactamase families and additional functional subgroups has been incorporated into the framework, as new variants are consistently being discovered (Table 1). In previous classifications, enzymes have been categorized based on their capability to inactivate specific *β*-lactam drugs and *β*-lactamase inhibitors [28].

#### 3.1.1. Group 1

These enzymes, known as cephalosporinases, belong to Class C. They are found in the chromosomes of various Enterobacteriaceae and a few other organisms [29]. Their activity is significantly higher when cephalosporins are present compared to benzylpenicillin, and they generally resist inhibition by clavulanic acid while remaining active in the presence of certain cephamycin-like compounds such as cefoxitin. These enzymes have a notably high affinity for aztreonam, with Ki (inhibition constant) values as low as 1–2 nM, which distinguishes them from Class A cephalosporinases [30, 31]. In many organisms, such as *Pseudomonas aeruginosa*, *Citrobacter freundii*, *Serratia marcescens*, and *Enterobacter cloacae*, the expression of AmpC is typically low. However, it can be induced when these bacteria are exposed to certain *β*-lactams, including amoxicillin, ampicillin, imipenem, and clavulanic acid [29, 32–34].

#### 3.1.2. Group 2

Functional Group 2 consists of S*β*Ls, encompassing molecular Classes A and D (Table 1). This group represents the largest category of *β*-lactamases, largely due to the growing detection of extended-spectrum *β*-lactamases (ESBLs) over the past two decades [6].

#### 3.1.3. Group 3

M*β*Ls form a unique subgroup of *β*-lactamases, distinguished by their specific structural and functional characteristics. The enzymes in clinical isolates are often coexpressed with other types of *β*-lactamases [35]. M*β*Ls are distinct from other *β*-lactamases in that, structurally, they need a Zn ion in their active site. They are also known to have a functional ability to hydrolyze carbapenems, with some S*β*Ls also gaining this ability. M*β*Ls have weak hydrolytic activity toward monobactams and have poor affinity for them compared to S*β*Ls [36]. Additionally, they are not inhibited by clavulanic acid or tazobactam but can be blocked by metal ion chelators such as EDTA, dipicolinic acid, and 1,10-phenanthroline [35].

## 4. M*β*L Overview

**Figure 3:**
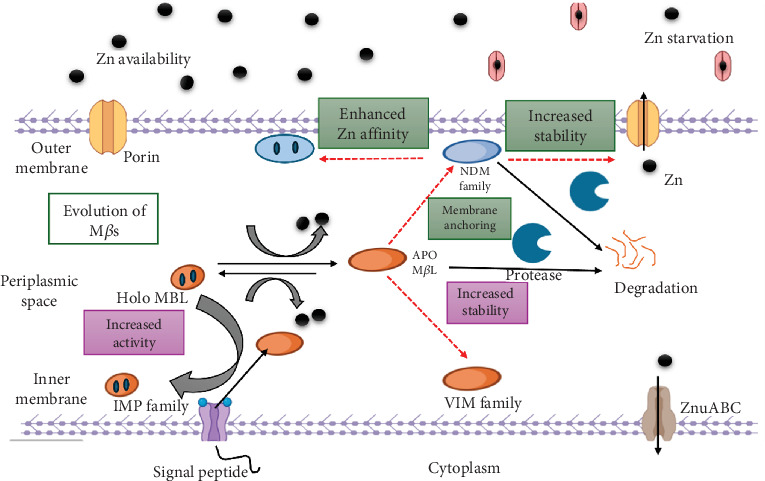
Mechanism of M*β*L evolution in Gram-negative bacteria.

M*β*Ls were initially discovered in 1966 in *Bacillus cereus*, where it was shown that metal chelators such as EDTA could inhibit the enzyme's cephalosporinase activity [37]. The evolution of M*β*Ls in Gram-negative bacteria is a complex process driven by selective pressure from antibiotic use, genetic adaptability, and HGT [38]. One of the primary drivers of M*β*L evolution is HGT, where M*β*L genes, such as those encoding New Delhi metallo-*β*-lactamases (NDMs), Verona integron-encoded metallo-*β*-lactamases (VIMs), and imipenemase (IMP) enzymes, are transferred between bacteria via mobile genetic elements like plasmids, transposons, and integrons (Figure 3) [39]. These factors allow the easy spread of resistance between various bacterial species. The blaNDM-1 gene that encodes for the NDM is usually located on plasmids that can be transferred easily among Enterobacteriaceae, *Pseudomonas*, and *Acinetobacter* spp. [40]. Furthermore, gene duplication and divergence contribute to M*β*L evolution because duplicated genes may accumulate mutations that increase enzyme activity or expand substrate specificity [41]. These enzymes are dependent on divalent cations, typically Zn, for hydrolysis activities. The longest M*β*Ls are approximately 250 amino acid residues [42]. M*β*Ls target a carbon (C)–nitrogen (N) bond of the *β*-lactam ring. This is a covalent bond between a C atom and a N atom, which is typical in organic compounds like amines, amides, and nitriles. In *β*-lactam antibiotics, the C-N bond is an essential component of the *β*-lactam ring structure [43]. The originally detected M*β*Ls were located on chromosomes and produced by nonclinical organisms, that is, *Flavobacterium odoratum*, *Bacillus cereus*, and *Legionella gormanii* [44]. Many clinical isolates of *Burkholderia cepacia* produce PCM-I, an inducible metalloenzyme that hydrolyzes carbapenems/imipenem [45]. It has been discovered that a low percentage of *B. fragilis* isolates produce the chromosomal metalloenzyme CfiA/CcrA, which confers imipenem resistance [46].

### 4.1. Categorization and Variability in M*β*Ls

M*β*Ls are categorized into various groups based on their genetic and biochemical properties. These enzymes have been categorized based on the Ambler classification (Class B) and the Bush–Jacoby classification, which differentiates *β*-lactamases by their amino acid compositions and substrate characteristics [27, 28]. M*β*Ls are divided into three subclasses based on differences in catalytic amino acids, zinc-binding ligands, zinc composition, loop structures, and substrate profiles. Some of the well-known acquired M*β*Ls, such as IMP, NDM, and VIM, are part of Subclass B1 [47].

These enzymes can break almost all *β*-lactam antibiotics, excluding monobactams (aztreonam), and are similar to other enzymes of Subclasses B1 and B3. In contrast, the *Aeromonas* spp. M*β*Ls categorized as CphA (Subclass B2) show a narrow spectrum of activity primarily against carbapenems. Microbes expressing M*β*Ls are also resistant to S*β*Ls inhibitors like clavulanic acid, sulbactam, tazobactam, and avibactam [48].

Most acquired Subgroup B1 M*β*Ls (Table 2) are derived from the initial discovery of VIM and NDM. In the 1990s, clinical isolates of *P. aeruginosa* and *S. marcescens* were recognized as a source of the first acquired M*β*L (IMP; IMP-1) in Japan [65]. Currently, the IMP-1 family has more than 85 sequence variants [66]. In 1997, the first VIM enzyme was discovered in *P. aeruginosa* [67]. In 2008, NDM was detected in *K. pneumoniae* and *E. coli* isolates from a patient who migrated from New Delhi, India, to Sweden. NDM has since become the most prevalent M*β*L [68]. The Subclass B3 M*β*L is present in *S. maltophilia,* and it differs from other M*β*Ls because it contains four identical subunits along with S*β*L [69]. This combination provides defense against nearly all *β*-lactams. *M. elizabethkingia* has a B1 enzyme (*BlaB)* and a B3 type (*GOB*), which are two chromosomal M*β*Ls that contribute to resistance [70].

### 4.2. Catalytic Mechanism of M*β*Ls

**Figure 4:**
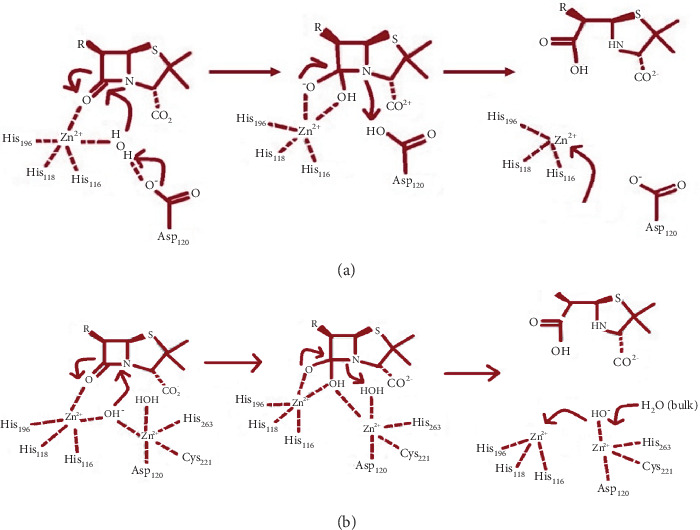
M*β*L mechanism of action for both monozinc and dizinc forms (a) Monozinc. (b) Dizinc.

In 1998, Bounaga et al. described a mechanism for the Subclass B1 enzymes [71]. The Zn ion coordinates with a water molecule and the histidine amino acid residues His116, His118, and His196. The lowering of the pK_a_ (acid dissociation constant) of the water molecule is caused by the coordination of the Zn^2+^ with the water molecule and the surrounding amino acid residues (His116, His118, and His196). The zinc ion allows it to act as a Lewis acid, existing as a hydroxide ion at neutral pH. This hydroxide ion attacks the C atom of the carboxyl group of antibiotics and creates a tetrahedral intermediate that is stabilized by the zinc ion. Subsequently, Asp120 deprotonates the hydroxyl group, resulting in a negatively charged intermediate that stabilizes the zinc ion. In the final step, Asp120 provides a proton to the N atom of the *β*-lactam ring, promoting ring opening as shown in Figure 4a [72].

For the dizinc forms of M*β*Ls, a cephalosporin hydrolysis mechanism is proposed as shown in Figure 4b. The protonation of a ring-opened intermediate containing anionic N serves as the rate-limiting step. Nitrocefin is a chromogenic cephalosporin substrate widely used to detect *β*-lactamase activity. This hydrolysis mechanism is considered uncommon due to its unique structure, particularly the presence of a styryl group with two nitro groups. Nitrocefin contains a *β*-lactam ring fused to a dihydrothiazine ring, like other cephalosporins, but the styryl group attached to the *β*-lactam ring, with nitro groups at the ortho and para positions, distinguishes it. When *β*-lactamases hydrolyze nitrocefin, the *β*-lactam ring is opened through a nucleophilic attack by a hydroxide ion (OH^−^), often generated by zinc ions in M*β*Ls [73, 74]. The styryl group, with its two nitro groups, plays a critical role in enhancing the stability and reactivity of nitrocefin [73].

For Subclass B2, Asp124 and His118 generate nucleophilic hydroxide. This ion attacks the carbonyl group of the *β*-lactam. His196 is responsible for activating the reaction [75]. A major bond rotation occurs after the *β*-lactam bond breaks, and the oxygen atom of the 6-hydroxyethyl derivative, an intermediate structure formed during the hydrolysis of *β*-lactam antibiotics, such as penicillins or cephalosporins, by *β*-lactamase enzymes, performs a nucleophilic attack on C3 with immediate proton transfer to C2 [76].

### 4.3. Epidemiology of M*β*Ls

**Figure 5:**
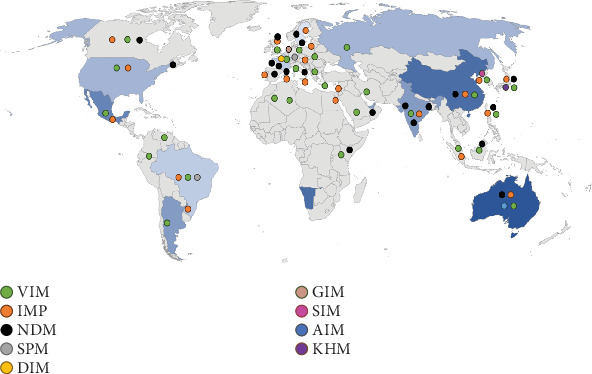
Global distribution of metallo-*β*-lactamases.

Over the past few decades, these enzymes have evolved into a global concern with acquired genes in clinical isolates of the United States and Asia (Figure 5). The most prevalent types include IMP, NDM, VIM, SIM, SPM, and GIM [77, 78]. The first rapid spread of an acquired M*β*L gene was reported in Australia. Bacteria that showed a mutant of IMP-1 and IMP-4 also caused the infection [79, 80]. This form of IMP-1 was identified in Chinese and Hong Kong isolates of *Citrobacter youngae* and *Acinetobacter* spp. [81]. Different Gram-negative species were found to harbor these genes in hospital-acquired isolates. International travelers from other regions were likely responsible for the importation of acquired strains into Australia. These genes interact with other genes on mobile DNA elements and further enhance the dissemination of infection [82]. Scientists uncovered that a minority of acquired strains are carbapenem-resistant [83]. In Europe and North America, M*β*L-producing Enterobacterales have been increasingly reported in hospital-acquired infections, especially in intensive care units (ICUs) [84]. In Asia, the NDM has been found in India, Pakistan, and China, where it is endemic in both clinical and environmental environments [85]. The Middle East and South America have also documented an increase in M*β*L-producing bacteria, especially among *P. aeruginosa* and *A. baumannii* isolates [86]. The Centers for Disease Control and Prevention (CDC) and the European Centre for Disease Prevention and Control (ECDC) have ranked M*β*L-producing bacteria as a critical priority of pathogens that require immediate research and development efforts [87, 88].

The economic costs of M*β*L infections are high given their correlation with escalated hospitalization expenses, extended hospital stays, and increased mortality. Research has indicated that M*β*L-producing bacterial infections lead to longer hospital stays (20–30 days on average) and a higher financial burden as compared to drug-susceptible infections [89]. In low and middle-income nations (LMICs), M*β*L infection treatment is especially a daunting task with restricted access to new antibiotics, inadequate infection control measures, and less-developed diagnostics. This has created a vicious cycle of misuse of antibiotics, further promoting resistance and creating the demand for costlier last-resort options like cefiderocol or colistin [90]. The social effect of M*β*L infections is also noteworthy. Most of the cases are in immunocompromised individuals, the elderly, and in ICU units, raising risks for morbidity and mortality. The psychological and economic burden on families from extended treatment courses and the need for isolation precautions also complicate the situation [91].

## 5. Structure and Catalytic Functions of M*β*Ls

**Figure 6:**
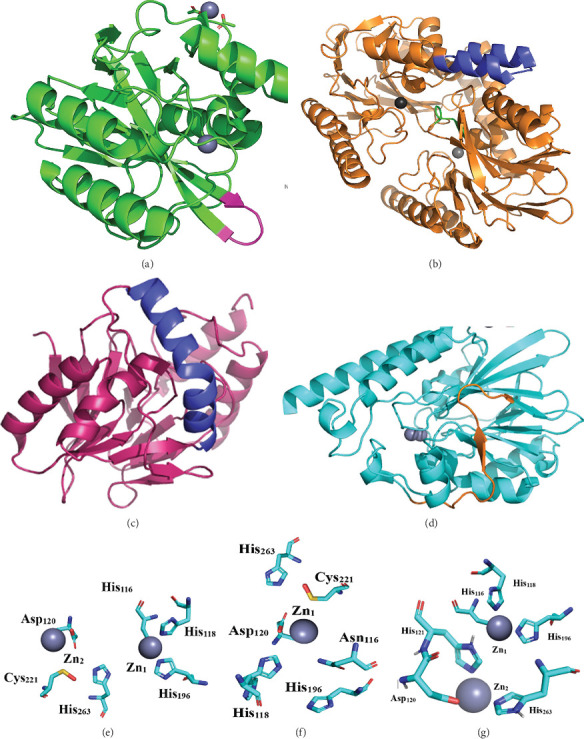
3D structures of M*β*Ls from different subclasses—B1, B2, and B3—and zinc-binding amino acid residues in different subclasses of M*β*Ls. (a) Subclass B1 VIM-2 shows the flexible loop extended across residues 61–65, with the position highlighted in magenta. (b) Subclass B1 enzyme SPM-1, the loop at residues 61–65 shown in green and the *α*3–*α*4 region in blue. (c) Subclass B2 CphA enzyme featuring an elongated *α*3 helix (residues 140–161) represented in blue. (d) Subclass B3 AIM-1 enzyme loop between residues 151 and 166 is shown in orange. (e) Crystal structures of B1 enzymes reveal two zinc-binding sites (Zn1 and Zn2). The Zn1 site is coordinated by three histidine residues (His116, His118, and His196), while the Zn2 site is coordinated by aspartic acid (Asp120), cysteine (Cys221), and histidine (His263). (f) In subclass B2 enzymes, the active site contains a single zinc ion and (g) two zinc ions in the active site of Subclass B3.

The single-zinc structure of *Bacillus cereus* metallo-*β*-lactamase II (BcII) was the first three-dimensional structure to be solved [92]. Subsequently, the structure of the Subclass B2 enzyme CphA [75], BcII dizinc form [92], VIM-2 and SPM-1 [93], IMP-1 [94], and subclass B3 enzymes FEZ-1 and AIM-1 was available [95]. A loop (61–65) exists in the N-terminal domain of B1 enzymes capable of binding to the hydrophobic side chains of molecules like substrates or inhibitors (Figure 6a). This loop shows maximum flexibility in the enzyme's natural state. When the inhibitor or substrate enters the active region, the loop blocks the inhibitor molecules. The R-group of Trp64 residue interacts with the substrate's hydrophobic side chain [37]. Additionally, when inhibitors attach to the loop, it becomes stable and changes the active site cleft into an opening shaped as a narrow tube. The removal of this loop is known as the flap. Apart from imipenem, the flap reduces the enzyme's activity by minimizing the substrate–enzyme interaction [96]. The lack of an active site loop barely affects the kinetic parameters of the carbapenems. Carbapenems are different from other *β*-lactam antibiotics because of the absence of bulky aromatic ring alterations [97].

The Subclasses B2 and B3 do not have the 61–65 flap. The Subclass B2 M*β*L (CphA) has the extended *α*3 helix (Arg140–Leu161) near the active region cleft (Figure 6c). The helix contains a twist at Trp150 that allows *α*3 to track the curvature of the protein's surface and helps in binding the carbapenem substrate. Consequently, CphA has a distinct active site, accounting for the enzyme's minimal activity profile [98]. Subclass Bl SPM-1 enzymes do not have the mobile loop (residues 61–65) in their structure. In SPM-1, a compact turn made up of five amino acid residues separates *β*3 and *β*4 strands (Figure 6b). Additionally, an extended *α*3–*α*4 region contains a central insertion of 24 amino acids. Deletion of this insertion has a minimal effect when a variety of *β*-lactam antibiotics bind and are hydrolyzed [93]. The Subclass B2 CphA enzyme shares both SPM-1 structural features. In addition, the Subclass B2 enzyme IMP-1 (35.5%), ImiS (32.2%), and CphA (32.1%) have the highest similarities when all M*β*Ls were compared with the SPM-1 sequence [99].

In Subclass B3 M*β*Ls, a loop composed of residues 151–166, which connects *α*3 and is located near the active region *β*7, is mobile, as shown in Figure 6d [93, 100].

## 6. Diagnostic Tools for *β*-Lactamases

Several diagnostic tools have been developed to detect *β*-lactamases and their variants in pathogens such as *E. coli*, *K. pneumoniae*, *Haemophilus influenzae*, and *A. baumannii* [101, 102]. The nitrocefin test is a simple colorimetric assay that detects *β*-lactam hydrolysis [103]. The modified Hodge test (MHT) is used to identify carbapenemase activity in Enterobacteriaceae, whereas the Carba NP test provides a rapid and specific method for detecting carbapenemase production [104]. Molecular techniques, such as PCR and loop-mediated isothermal amplification (LAMP), permit the identification of *β*-lactamase genes like blaCTX-M, blaKPC, and blaNDM [105]. Sophisticated methods such as MALDI-TOF mass spectrometry identify the products of *β*-lactam hydrolysis, enabling the rapid identification of resistant strains [102]. Whole-genome sequencing (WGS) is a valuable tool for surveillance and epidemiological investigation of AMR [106]. Conventional phenotypic techniques like disk diffusion and Etest continue to be used extensively for determining antibiotic susceptibility [107]. Moreover, *β*-lactamase inhibitor tests with agents like clavulanic acid assist in establishing resistance mechanisms [108]. As shown in Table 3, these tests play an essential role in the direction of effective antibiotic therapy and in preventing the spread of *β*-lactamase-producing organisms in healthcare settings [107].

## 7. Inhibitors of M*β*L-Containing Bacterial Infections

The therapeutic strategies for their inhibition have focused on three major approaches: the development of synthetic boronate-based inhibitors, the use of metal chelators, and the repurposing of clinically approved drugs. In addition, natural product-derived scaffolds and novel dual-action molecules have emerged as promising alternatives [112].

### 7.1. Boronate-Based Inhibitors

Boronate-based inhibitors represent the most clinically advanced candidates against M*β*Ls. Taniborbactam (VNRX-5133) is a cyclic boronate compound currently undergoing Phase 3 clinical trials in combination with cefepime for the treatment of complicated urinary tract infections. It exhibits broad-spectrum activity not only against S*β*Ls but also against NDM-, VIM-, and IMP-type metallo-*β*-lactamases, demonstrating significant clinical promise [113]. Xeruborbactam (QPX7728), another cyclic boronate inhibitor, exhibits an even broader inhibitory spectrum and maintains stability in the presence of efflux pumps and porin loss, which are common mechanisms of resistance [114]. Zidebactam and WCK 5153 are novel bicyclo-acyl hydrazide (BCH) agents that have previously been shown to act as *β*-lactam enhancer (BLE) antibiotics in *P. aeruginosa* and *A. baumannii*. Zidebactam, a PBP2-binding BLE, provides partial inhibition of M*β*Ls in combination regimens, highlighting the versatility of boronate-based approaches [115].

### 7.2. Metal Chelators

Since zinc ions are essential for the catalytic activity of M*β*Ls, zinc chelation has been explored as a means of enzyme inhibition. Traditional chelators, such as EDTA, demonstrate strong in vitro activity but are unsuitable for systemic therapy due to their toxicity [116]. A novel fluorescent probe (CE-HF) enabled the identification of ethylenediamine tetra(methylene phosphonic acid) or EDTMP as a potent, broad-spectrum inhibitor. EDTMP restored meropenem activity by zinc chelation, showing strong synergistic antibacterial effects against resistant pathogens [117]. Advanced chelators such as TPEN (N,N,N⁣′,N⁣′-tetrakis(2-pyridylmethyl) ethylenediamine) and NOTA (1,4,7-triazacyclononane-1,4,7-triacetic acid) have shown promise in restoring the efficacy of meropenem both in vitro and in animal models [118, 119]. Dipicolinic acid and Ca-EDTA have also been tested with encouraging results, indicating that the rational design of selective chelators could lead to clinically viable inhibitors [120]. These studies confirm the feasibility of targeting the zinc-dependent catalytic mechanism of M*β*Ls, although safety and pharmacokinetics remain major barriers.

### 7.3. Repurposed Drugs

Repurposing of existing FDA-approved drugs provides a practical pathway for rapid clinical translation of M*β*L inhibitors. Proton pump inhibitors such as pantoprazole and omeprazole have been reported to inhibit M*β*Ls by chelating zinc, in addition to reducing biofilm formation and downregulating resistance genes like blaNDM and blaVIM. This dual action enhances the effectiveness of meropenem against resistant isolates [121]. Similarly, trifluoromethylated captopril analogues were developed as promising M*β*L inhibitors, with the most active derivative showing strong inhibitory activity against NDM-1. Several compounds also restored the efficacy of meropenem, markedly reducing resistance in *E. coli* strains carrying NDM-1, VIM-2, and IMP-26 [122]. However, the clinical applicability of captopril derivatives has been limited by issues of toxicity and instability. Nevertheless, these findings highlight the potential of drug repurposing strategies to expand therapeutic options against multidrug-resistant organisms [123].

### 7.4. Natural Product–Derived Inhibitors

Natural products offer structurally diverse scaffolds for M*β*L inhibition. Aspergillomarasmine A (AMA), a fungal metabolite, has demonstrated the ability to remove zinc from the active site of NDM and VIM enzymes, restoring carbapenem activity in preclinical infection models [124]. AMA restored meropenem activity against M*β*L-producing strains, but resistance persisted in bacteria coharboring M*β*Ls and S*β*Ls. A triple combination of AMA, avibactam, and meropenem showed strong efficacy, particularly against Enterobacterales clinical isolates with multiple *β*-lactamases [125]. Plant-derived flavonoids, including quercetin and baicalein, exhibit moderate M*β*L inhibition but also provide additional antiquorum-sensing and antibiofilm activities, which could be advantageous in controlling chronic infections [126, 127]. Such multifunctional properties may be crucial for the development of next-generation inhibitors.

### 7.5. Dual-Action Molecules and Combination Therapies

In addition to standalone inhibitors, combination strategies have gained attention for overcoming M*β*L-mediated resistance. Aztreonam combined with avibactam is now used clinically for infections caused by coproducing organisms, as aztreonam is stable to hydrolysis by M*β*Ls while avibactam neutralizes coproduced S*β*Ls [128]. A recent study has shown that quinazolinone analogs (QAs), a dual-action molecule or zinc chelator, have been described as an inhibitor that not only blocks NDM-1 activity but also disrupts biofilms, thereby enhancing the bactericidal effects of carbapenems [129].

### 7.6. Antibiotics and Their Combinations for M*β*L Inhibition

M*β*Ls are resistant to nearly all known inhibitors and inactivators of S*β*Ls. The active site of M*β*Ls shares less than one-third of sequence similarities between the subclasses, which poses a significant challenge in designing inhibitors like taniborbactam that inhibit NDM and VIM enzymes but fail to inhibit IMP enzymes [130]. Since no clinically approved “pure” M*β*L inhibitor is available, therapeutic approaches rely mainly on antibiotic combinations. These strategies include combining antibiotics with *β*-lactamase inhibitors, zinc chelators, or synergistic antimicrobials, as shown in Table 4. One of the most well-studied approaches is the combination of aztreonam with avibactam, which has shown remarkable efficacy against NDM-, VIM-, and IMP-producing Enterobacterales and *P. aeruginosa* [143]. In this regimen, avibactam neutralizes Class A, C, and D S*β*Ls, while aztreonam remains unaffected by M*β*L hydrolysis, resulting in a powerful synergy. This combination has been proven highly effective both in vitro and in clinical reports, providing a valuable therapeutic option for infections caused by NDM-positive carbapenem-resistant Enterobacterales (CRE) [131].

A related strategy employs aztreonam in combination with clavulanate or tazobactam, which target NDM-harboring CRE and *P. aeruginosa*. Here, clavulanate or tazobactam protects aztreonam from degradation by ESBLs or AmpC *β*-lactamases, while aztreonam maintains stability against M*β*Ls. Several studies report potent synergistic activity and successful clinical outcomes, although the regimen is somewhat complex and lacks large-scale clinical trial validation [132].

Another approach is the use of double carbapenem therapy, such as ertapenem with meropenem, which exploits differential affinities for carbapenemases, although clinical evidence remains limited [133]. Similarly, the combination of cefiderocol with avibactam offers promise against NDM-, VIM-, and IMP-producing Enterobacterales. In this case, cefiderocol utilizes iron transport pathways to bypass resistance mechanisms, while avibactam inhibits coproduced S*β*Ls. Studies demonstrate significant in vitro synergy, but resistance may emerge due to altered iron uptake pathways or M*β*L variants [134].

More complex regimens, such as meropenem combined with vaborbactam and aztreonam, have been reported as salvage therapies for Enterobacterales coproducing NDM and KPC. Here, vaborbactam targets KPC, aztreonam avoids MBL hydrolysis, and meropenem provides bactericidal activity, resulting in strong synergy [135, 136].

Alternative adjunctive strategies include proton pump inhibitors such as pantoprazole or omeprazole combined with meropenem, which may interfere with efflux pump–mediated resistance [121]. Traditional last-line options such as polymyxin B or colistin in combination with carbapenems target XDR *P. aeruginosa* and *A. baumannii*. Polymyxins disrupt bacterial membranes, enhancing carbapenem penetration, and in vitro synergy has been observed. Nevertheless, these regimens are hampered by high nephrotoxicity and neurotoxicity, as well as inconsistent clinical outcomes [137, 138]. Likewise, tigecycline combined with colistin or carbapenems has been explored against CRE and M*β*L producers, including *K. pneumoniae* and *A. baumannii*. This strategy merges ribosomal inhibition by tigecycline with membrane disruption or *β*-lactam activity [139, 140].

## 8. Emerging Resistance to New Inhibitors

According to recent studies, resistance is evolving against promising novel *β*-lactam inhibitor combinations. Taniborbactam is highly active against NDM and VIM M*β*Ls; it shows limited efficacy against IMP variants due to active site structural differences [144]. By interrupting important electrostatic interactions in the active site of the enzyme, variants such as NDM-9, which are altered from NDM-1 by a single point substitution of Glu152Lys, exhibit higher resistance to taniborbactam, resulting in ineffective binding and substantially reduced inhibition [145]. Point mutations in zinc-coordinating amino acids or flexible loops can rapidly diminish inhibitory activity, based on analogous mechanisms observed in produced VIM variants. These findings emphasize the importance of continued surveillance, the optimization of structure-guided inhibitors, and the potential use of combination therapies to delay the onset of resistance [144].

Another cyclic boronate dual inhibitor, xeruborbactam, has a broader activity profile of M*β*L inhibition, including IMP-type enzymes that are not inhibited by taniborbactam, but it still retains inhibitory activity against NDM-9 [114]. Some IMP variants (IMP-6, IMP-10, IMP-14, and IMP-26) are also resistant to inhibition by xeruborbactam because of structural mutations to binding (e.g., Ser262Gly in IMP-6, Phe substitution in IMP-10). Efflux pumps may also diminish the efficacy of xeruborbactam in *P. aeruginosa*, such as the MexAB-OprM system significantly decreasing activity [146].

In a similar mechanism, cefiderocol, a siderophore-cephalosporin that depends on bacterial iron transport mechanisms to bypass porin-based resistance, has shown evidence of heteroresistance in *A. baumannii* and Enterobacterales, via tonB-dependent receptor (piuA) mutations and *β*-lactamase gene amplification [141]. These findings have shown that even “next-generation” antibiotics can be confronted with the emergence of resistance quickly, thus highlighting the importance of in vitro experimental studies to continuously evaluate the antibiotics' efficacy and monitor the emergence of drug-resistant bacteria and spread and highlighting the importance of combining antibiotic treatments [142].

Despite next-generation *β*-lactamase inhibitor combinations, more reports of effective combination therapy resistance are being seen. Meropenem–vaborbactam resistance in KPC-producing *K. pneumoniae* has been linked to porin loss (OmpK35/OmpK36) and amplification of the blaKPC gene, which decreases drug absorption [147]. By preventing inhibitor binding with single amino acid changes, mutants such as NDM-9 in *E. coli* and VIM-83 in *Enterobacter cloacae* have been shown to exhibit high-level resistance to cefepime–taniborbactam. Overexpression of AmpC and MexAB-OprM efflux pump in *P. aeruginosa* reduces susceptibility to imipenem–relebactam and even to cefiderocol, while mutations or loss of TonB-dependent siderophore receptors (cirA, fiu) in *A. baumannii* hinders cefiderocol activity [148]. Through the combined effects of PBP modification and enzyme production, ceftazidime–avibactam and aztreonam resistance may also be acquired by dual carbapenemase producers like NDM + OXA-48 in *K. pneumoniae*. Together, these studies illustrate how several adaptive mechanisms, such as efflux, target site modifications, porin recasting, and gene amplification, act in concert to weaken even the best combination regimens [149].

### 8.1. Mechanistic Insights Into *β*-Lactamase Mutations

Resistance development often arises from single amino acid mutations, which affect the active sites of *β*-lactamases. In Class A *β*-lactamases, mutations at Positions 104, 164, 238, and 240 in temoneira *β*-lactamases (TEMs) can broaden the spectrum of substrates while reducing susceptibility toward inhibitors like clavulanic acid [9]. Similarly, M*β*Ls with structural remodeling caused by rearrangement of loops or substitutions of residues that coordinate zinc can lessen the binding efficiency of inhibitors without affecting catalytic function [37]. Mechanistic insight is valuable for rational drug design as it provides predictable locations of mutations (hotspots) that facilitate inhibitor escape [38].

### 8.2. Structure-Guided Drug Design and Natural Inhibitors

Advances in structural biology have aided the design of new scaffolds for inhibitors. For example, *β*-lactamase inhibitor protein (BLIP) from *Streptomyces* matures picomolar inhibition (Ki ≈ 0.6 nM) against TEM-1 enzymes, and the protein is amenable to engineering with phage display and has been targeted toward additional resistant *β*-lactamases. These structure-based models illustrate the potential for custom-designed inhibitors to combat developing variants in *β*-lactamases [48]. Additionally, the aim of dual-action inhibitors to target both S*β*Ls and M*β*Ls with active site zinc chelators attached to a *β*-lactam scaffold is also being designed and shows promising effects in preclinical models [150]. Furthermore, endolysins and depolymerases derived from bacteriophages have developed as novel potential antimicrobials. These enzymes specifically degrade bacterial peptidoglycan or capsule polysaccharides, with relatively little likelihood of developing resistance. Enzyme phage, combined with *β*-lactams, has been shown to increase bacterial clearance, kill biofilms, and restore susceptibility in multidrug-resistant pathogens. These “enzybiotics” utilize completely different mechanisms of action that circumvent traditional resistance pathways. Their use as additional therapeutic agents helps augment small molecule inhibitors [151].

## 9. Challenges in the Development of Novel Therapeutics Against M*β*Ls

The development of new drugs to combat infections caused by M*β*Ls presents several significant challenges, primarily due to the unique biochemical properties of M*β*Ls and their ability to rapidly evolve resistance mechanisms [152]. Below are a few main challenges involved in developing effective therapies against M*β*L-producing pathogens.

### 9.1. Structural Complexity and Diversity

The variability in different classes of these enzymes makes the drug ineffective against all variants. To design a broad-spectrum inhibitor that targets the diverse structural features of M*β*Ls is a significant challenge. Additionally, the active site can endure structural changes that complicate the growth of inhibitors that can bind effectively and inhibit enzyme function across different strains [153].

### 9.2. Resistance Mechanisms and Coresistance

M*β*Ls often coexist alongside other resistance mechanisms, including efflux pumps, altered porin channels, and other *β*-lactamases. Simultaneously, coresistance mechanisms enable bacteria to resist multiple classes of antibiotics. Inhibitors like avibactam or EDTA are effective against M*β*Ls in lab settings, yet they are frequently ineffective in clinical scenarios due to other resistance mechanisms. These mechanisms are crucial and highly challenging to develop new drugs that target multiple resistance pathways [148].

### 9.3. Targeting Zinc in Active Sites

These enzymes have zinc ions at their active sites to facilitate the hydrolysis of *β*-lactam antibiotics. Developing inhibitors that effectively chelate these zinc ions without disrupting normal cellular functions remains a difficult task. Designing molecules that target the M*β*L active site zinc ions without affecting other essential zinc-dependent enzymes in the human body requires highly selective and nontoxic compounds, which are difficult to discover and optimize [154].

### 9.4. Rapid Evolution and Adaptability

Bacteria can rapidly evolve and adapt to new antibiotics, particularly through HGT mediated by plasmids. This genetic flexibility enables M*β*L-producing bacteria to acquire new resistance mechanisms. As a result, any new drug is less effective in treating bacterial infections. Developing drugs with resistance-modifying properties combined with other inhibitors to prevent resistance remains a challenge [15].

### 9.5. Complex Drug Development Pathway and High Costs

Drug development is a time-consuming and high-cost procedure, often taking over a decade from discovery to market. Further, the process is complicated by the need for effective, safe, affordable, and easily scalable compounds [155, 156]. Due to the high cost of developing medications and the unpredictable nature of M*β*L resistance, pharmaceutical companies may stop investing in this area. Research on vaccine development and its implementation against AMR could be considered an alternative approach to combat such deadly drug-resistant infections [157]. Some of these financial difficulties could be addressed by government funding and public–private collaborations.

## 10. Conclusion and Future Perspectives

The growing threat of *β*-lactam resistance, particularly due to *β*-lactamases, has become a major concern for global health. M*β*Ls pose a significant challenge to manage antibiotic resistance because of their ability to inactivate a wide range of *β*-lactam antibiotics, including carbapenems, often considered as the new-generation antibiotics for several bacterial infections. The spread of M*β*L-producing bacteria is responsible for the inhibition of new-generation therapeutics. M*β*Ls degrade the molecular structure of antibiotics by breaking the *β*-lactam ring with zinc ions. Overcoming antibiotic resistance requires more effective treatments. Trend analysis of resistance, direct public health initiatives, and more worldwide awareness are essential to combat the resistance. Accurate and quick diagnostics can reduce the overuse of broad-spectrum antibiotics by ensuring prompt and focused therapy. Antimicrobial stewardship initiatives are essential for the public and healthcare professionals, as they are critical for disseminating the rational use of antibiotics amongst the patient population. To understand the role of antibiotics properly, a deeper understanding of M*β*L structural biology and the interplay between different resistance mechanisms is essential. The integration of combination therapies targeting multiple resistance mechanisms and innovative approaches will shape the future of M*β*L management. Future research should prioritize multidisciplinary strategies that combine microbiology, bioinformatics, pharmacology, and public health initiatives to mitigate the global threat of M*β*L-producing pathogens. This information could drive the design of more potent inhibitors or combination therapies capable of overcoming not only M*β*Ls but also other coexisting resistance factors, such as efflux pumps and altered porins. Moreover, there is an urgent need for more comprehensive global surveillance to track the spread of specific M*β*L variants and monitor regional differences in resistance patterns, which would be helpful for targeted interventions and public health strategies.

## Figures and Tables

**Table 1:** *β*-Lactamases are classified based on systems developed by Bush et al. and Ambler (CA refers to clavulanic acid and TZB to tazobactam).

**Bush–Jacoby scheme**	**Ambler's classification**	**Primary antibiotics**	**Inhibited by**		***β*-Lactamase examples**	**References**
			**CA or TZB**	**EDTA**		
Group 1	C	Cephalosporins	No	No	*E. coli* AmpC	[11]
Group 1e	C	Cephalosporins	No	No	P99, ACT-1, CMY-2, FOX-1, MIR-1 GC1, and CMY-37	[11]
Group 2a	A	Penicillins	Yes	No	PC1	[11]
Group 2b	A	Penicillins, early cephalosporins	Yes	No	TEM-1, TEM-2, and SHV-1	[11]
Group 2be	A	Extended-spectrum cephalosporins, monobactams	Yes	No	TEM-3, SHV-2, CTXM-15, PER-1, and VEB-1	[11]
Group 2br	A	Penicillins	No	No	TEM-30, SHV-10	[11]
Group 2ber	A	Extended-spectrum cephalosporins, monobactams	No	No	TEM-50	[11]
Group 2c	A	Carbenicillin	Yes	No	PSE-1, CARB-3	[11]
Group 2ce	A	Carbenicillin, cefepime	Yes	No	RTG-4	[11]
Group 2d	D	Cloxacillin	Variable	No	OXA-1, OXA-10	[11]
Group 2de	D	Extended-spectrum cephalosporins	Variable	No	OXA-11, OXA-15	[11]
Group 2df	D	Carbapenems	Variable	No	OXA-23, OXA-48	[11]
Group 2e	A	Extended-spectrum cephalosporins	Yes	No	CepA	[11]
Group 2f	A	Carbapenems	Variable	No	KPC-2, IMI-1, and SME-1	[11]
Group 3a	B (B1)	Carbapenems	No	Yes	IMP-1, VIM-1, CcrA, and IND-1	[11]
	B (B3)	Carbapenems	No	Yes	CAU-1, FEZ-1	[11]
Group 3b	B (B2)	Carbapenems	No	Yes	CphA, Sfh-1	[11]

**Table 2:** Some examples of chromosomal and plasmid-associated M*β*Lase activity.

**M*β*L type**	**Subclass**	**Species**	**Enzyme(s)**	**References**
Chromosomal	B1	*Bacillus cereus*	BcII	[49]
		*Chryseobacterium indologenes*	IND	[50]
		*Elizabethkingia meningoseptica*	BlaB	[51]
		*Myroides odoratimimus*	MUS and MYO	[52]
		*Bacteroides fragilis*	CfiA/CcrA	[53]
	B2	*Aeromonas* spp.	CphA	[54]
	B3	*Stenotrophomonas maltophilia*	LI	[55]
		*Elizabethkingia meningoseptica*	GOB	[56]

Plasmid associated	B1		Verona integron-encoded metallo-*β*-lactamases (VIM)	[57]
			Imipenemase (IMP)	[58]
			Sao Paulo metallo-*β*-lactamases (SPMs)	[59]
			German imipenemase (GIM)	[60]
			KHM	[61]
			Dutch imipenemase (DIM)	[62]
	B3		*Serratia marcescens* metallo-*β*-lactamases (SMBs)	[63]
			Adelaide imipenemase (AIM)	[64]

**Table 3:** Conventional and advanced diagnostic tools for detection of the *β*-lactamases.

**Diagnostic tool**	**Pathogens detected**	**Detection method**	**Associated diseases**	**Examples of *β*-lactamases**	**References**
Nitrocefin test	*E. coli*, *K. pneumoniae*, *P. aeruginosa*, and *A. baumannii*	Chromogenic cephalosporin hydrolysis	UTIs, pneumonia, and bloodstream infections	TEM, SHV, and CTX-M	[103]

Modified Hodge test (MHT)	*K. pneumoniae*, *E. coli*	Carbapenemase activity detection using an indicator strain	Carbapenem-resistant infections	KPC, NDM	[104]

Carba NP test	*K. pneumoniae*, *P. aeruginosa*, and *A. baumannii*	pH colorimetric assay for carbapenem hydrolysis	Multidrug-resistant infections	KPC, VIM, IMP, and NDM	[109]

Molecular PCR assays	*E. coli*, *K. pneumoniae*, *P. aeruginosa*, and *Enterobacter* spp.	Detection of *β*-lactamase genes (e.g., blaCTX-M, blaKPC, and blaNDM)	Hospital-acquired infections, UTIs, and sepsis	CTX-M, KPC, OXA-48, and NDM	[105]

Loop-mediated isothermal amplification (LAMP)	*E. coli*, *K. pneumoniae*	Rapid isothermal amplification of resistance genes	UTIs, bloodstream infections	CTX-M, KPC	[110]

MALDI-TOF mass spectrometry	*Enterobacteriaceae*, *P. aeruginosa*	Detection of *β*-lactam hydrolysis products	Respiratory and bloodstream infections	Various *β*-lactamases	[101, 102]

Whole-genome sequencing (WGS)	Various multidrug-resistant bacteria	Identification of resistance genes	Surveillance of antibiotic resistance	CTX-M, KPC, NDM, and OXA-48	[111]

Disk diffusion and Etest	*E. coli*, *K. pneumoniae*, and *P. aeruginosa*	Antibiotic susceptibility testing	Various bacterial infections	TEM, SHV, and CTX-M	[107]

*β*-Lactamase inhibitor-based tests	*E. coli*, *K. pneumoniae*, and *P. aeruginosa*	Use of inhibitors like clavulanic acid to confirm *β*-lactamase production	Nosocomial infections, UTIs	TEM, SHV, and CTX-M	[108]

**Table 4:** Existing therapeutic approaches for M*β*L-producing and multidrug-resistant Gram-negative pathogens, summarizing drug combinations, mechanisms, clinical relevance, and key limitations.

**S. no.**	**Combination therapy (drug + inhibitor, combination of antibiotics)**	**Partner/strategy**	**Target organisms/enzymes**	**Mechanism of action**	**Key findings/clinical impact**	**Limitations**	**References**
1	Aztreonam + avibactam	*β*-Lactam + *β*-lactamase inhibitor	NDM-, VIM-, IMP-producing Enterobacterales, *P. aeruginosa*	Avibactam inhibits serine *β*-lactamases (A, C, and D); aztreonam resists M*β*L hydrolysis	Highly effective in vitro and clinical reports; overcomes NDM-positive CRE	Limited availability; resistance via porin loss or efflux	[131]

2	Aztreonam + clavulanate/tazobactam	*β*-Lactam + inhibitor	NDM-harboring CRE and *P. aeruginosa*	Inhibitors protect aztreonam from ESBL/AmpC; aztreonam avoids M*β*L hydrolysis	Potent synergy; several clinical successes	Limited clinical trial data; regimen complexity	[132]

3	Double carbapenem (ertapenem + meropenem)	Sequential dual carbapenem	KPC/M*β*L coproducing Enterobacterales	Ertapenem acts as suicide inhibitor, freeing meropenem for action	Some success in refractory infections	Limited, variable results; not widely adopted	[133]

4	Cefiderocol + avibactam	Siderophore cephalosporin + inhibitor	NDM-, VIM-, IMP-producing Enterobacterales	Cefiderocol enters via iron transport; avibactam blocks serine *β*-lactamases	Synergy shown in vitro; prevents cefiderocol resistance	Resistance via iron uptake mutations and MBL variants	[134]

5	Meropenem + vaborbactam + aztreonam	Triple regimen	NDM- and KPC-coproducing CRE	Vaborbactam inhibits KPC; aztreonam avoids M*β*L hydrolysis; meropenem provides kill	Synergistic activity; salvage option	Limited to case reports; not tested in trials	[135, 136]

6	Meropenem + pantoprazole/omeprazole	Drug repurposing (PPI)	NDM-, VIM-producing CRE	PPIs chelate Zn^2+^ at the M*β*L active site	Restores carbapenem activity in vitro	Safety concerns; not optimized for systemic use	[121]

7	Polymyxin B/colistin + carbapenems	Combination	XDR *P. aeruginosa*, *A. baumannii*	Polymyxins disrupt membranes, improving carbapenem penetration	Synergy reported: Salvage therapy in XDR infections	Nephro/neurotoxicity; inconsistent results	[137, 138]

8	Tigecycline + colistin/carbapenem	Combination	CRE, M*β*L-producing multidrug-resistant *E. coli*, carbapenem-resistant *A. baumannii* and *K. pneumoniae*	Ribosomal inhibition + membrane disruption/*β*-lactam synergy	Clinical salvage regimen reduces resistance emergence	Low serum levels limit use in bacteremia; toxicity	[139, 140]

9	Cefiderocol	Siderophore cephalosporin	NDM-, VIM-, IMP-producing CRE, *P. aeruginosa*	Evades hydrolysis; Trojan horse iron uptake	High activity against M*β*L producers; approved for use	Resistance via M*β*L variants, iron transporter mutations	[141]

10	Meropenem + QAs (quinazolinone analogs)	Novel small molecule inhibitor	NDM-1, IMP-1 producers	Zn^2+^ chelation + biofilm disruption	Strong in vitro synergy	Preclinical only; no clinical data	[129]

11	Fosfomycin + *β*-lactams	Synergistic combination	MBL-producing CRE	Fosfomycin weakens cell wall, enhancing *β*-lactam action	Demonstrated in vitro efficacy	Clinical data lacking	[142]

12	Taniborbactam (VNRX-5133) + cefepime	Boronate-based *β*-lactamase inhibitor	NDM-, VIM-, IMP-, KPC-producing Enterobacterales	Transition-state analog; inhibits both serine *β*-lactamases and MBLs	Phase 3 trials; broad-spectrum activity	Resistance is possible via mutations in the MBL active site	[113]

13	Xeruborbactam (QPX7728) + meropenem	Ultra-broad boronate inhibitor	M*β*Ls (NDM, VIM, and IMP), KPC, and OXA	Strong reversible inhibition of S*β*Ls and M*β*Ls	Preclinical data show potent synergy	Still experimental	[114]

14	TPEN/NOTA + meropenem	Synthetic Zn^2+^ chelators	NDM, VIM enzymes	Chelates Zn^2+^ from the active site, inactivating M*β*Ls	Restores carbapenem activity in vitro and in animals	Toxicity and nonspecific chelation issues	[118, 119]

15	Aspergillomarasmine A (AMA) + meropenem	Natural fungal metabolite + *β*-lactam	NDM, VIM	Chelates Zn^2+^ ions from M*β*L enzymes	Restored carbapenem efficacy in mouse infection models	Stability, delivery, and toxicity issues	[125]

16	Flavonoids (quercetin, baicalein) + *β*-lactams	Plant-derived polyphenols + antibiotic	NDM-, VIM-producing CRE, *P. aeruginosa*	Weak Zn^2+^ chelation; might disrupt QS and biofilm regulatory proteins	Synergy observed in vitro	Low potency; bioavailability limitations	[126, 127]

## Data Availability

Data sharing is not applicable to this article as no datasets were generated or analyzed during the current study.
